# Obtaining Axenic Amastigotes of *Leishmania* sp. from an ulcer lesion of a patient with leishmaniasis

**DOI:** 10.1016/j.idcr.2021.e01261

**Published:** 2021-09-04

**Authors:** Jesús Rojas-Jaimes, Marco Mesia-Guevara

**Affiliations:** a Facultad de Ciencias de la Salud, Universidad Privada del Norte, Lima, Peru; b Escuela de Medicina Humana, Universidad Científica del Sur, Lima, Peru

Leishmaniasis is a neglected and endemic disease in Peru that is difficult to diagnose and treat. The parasites could be obtained from an 18-year-old male patient with no history of previous disease, a resident of the district of Huepetuhe, Madre de Dios. Endemic site of leihsmaniasis, with a regular contour ulcer with raised edges of approximately 2 cm radius in the anterior area of the leg with an evolution of approximately 3 weeks. In our study, an approximate 14-day biphasic blood agar culture of promastigote was obtained from a scraping of an ulcer lesion from a patient with leishmaniasis diagnosed clinically and corroborated by microscopy with the presence of amastigotes stained with Giemsa at 1000X. These culture promastigotes were placed in Schneider liquid medium, supplemented with 20% fetal bovine serum and 10 000 U/10 mg/mL penicillin streptomycin at pH 4.7 and 35 °C, obtaining 100% axenic amastigotes at 7 days on average ( Figs. 1 and 2).

**Fig. 1:**
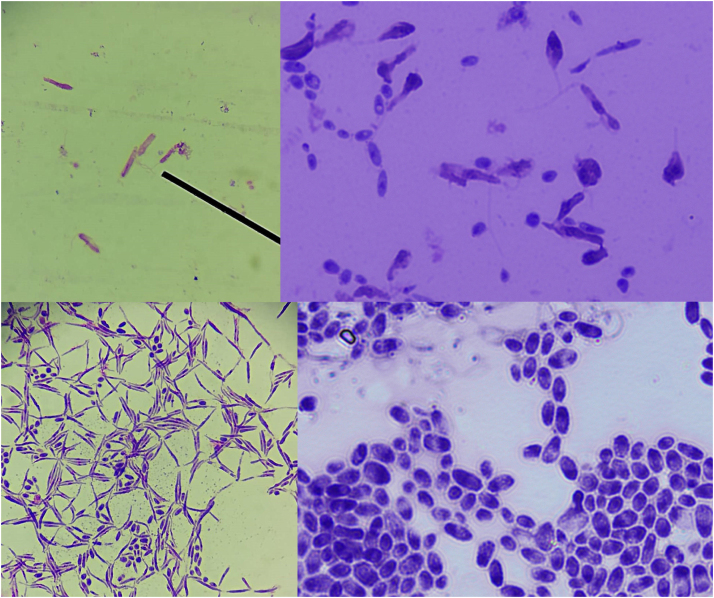
-From left to right from top to bottom Leishmania in axenic medium: promastigote at 0 days (400X), mixture between promastigote forms, pseudopromastigotes, pseudoaxenic forms at 3 days (1000X). Pseudopromastigotes and amastigotes forms at 5 days (400X). And axenic amastigotes at 7 days (1000X).

**Fig. 2:**
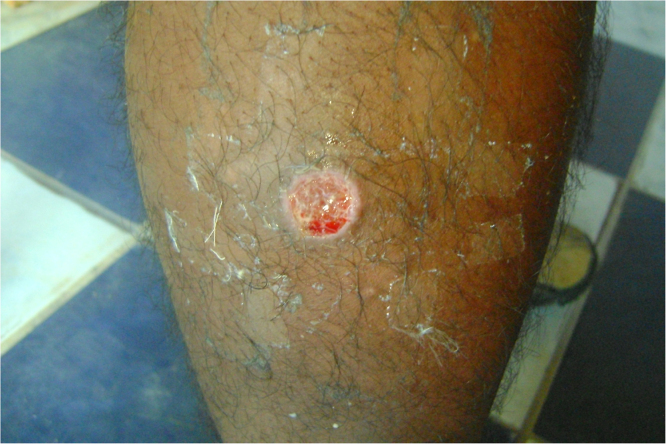
-Ulcer of patient with leishmaniasis.

From this it should be noted that the phenotype of the parasite is related to the transcriptomics of the parasite and therefore survival or susceptibility to the environment such as the host's immune system. Our study converted parasites of *Leishmania* sp. obtained from a biopsy of axenic amastigotes that could serve as models for transcriptomic studies and the testing of promising antiparasitic drugs.

## Conflict of interest

The authors declare that they have no conflict of interest.

